# 荷人肺癌小鼠皮下移植瘤模型的建立及其生物学特性初探

**DOI:** 10.3779/j.issn.1009-3419.2010.06.020

**Published:** 2010-06-20

**Authors:** 莹 卓, 一龙 吴, 爱林 郭, 思远 陈, 健 苏

**Affiliations:** 1 510080 广州，广东省心血管病研究所，广东省人民医院，广东省医学科学院 Guangdong Cardiovascular Institute, Guangdong General Hospital, Guangdong Academy of Medical Sciences, Guangzhou 510080, China; 2 510080 广州，广东省肺癌研究所，广东省人民医院，广东省医学科学院 Guangdong Lung Cancer Institute, Guangdong General Hospital, Guangdong Academy of Medical Sciences, Guangzhou 510080, China; 3 510515 广州，南方医科大学 Southern Medical University, Guangzhou 510515, China

**Keywords:** 肺肿瘤, 模型, NOD/SCID鼠, Lung neoplasms,, Disease models, NOD/SCID mice

## Abstract

**背景与目的:**

为了更好地研究肺癌的治疗方法，建立起可靠的动物评价模型迫在眉睫。本研究的目的是研究建立肺癌原代组织块小鼠移植瘤模型的成熟方法，观察移植瘤的肿瘤生物学特性，在建模方法及基本特征等方面来证明该肿瘤模型的合理性和科学性，以期为肿瘤研究提供更有效的实验动物模型。

**方法:**

取人新鲜完整肺癌组织块移植于NOD/SCID小鼠右侧前肢肩背部皮下，或经皮肺穿刺取得肿瘤小块移植于BALB/c裸小鼠肾包膜下。待皮下肿瘤增大，将其切下传代于裸小鼠右侧前肢肩背部皮下。观察移植瘤生物学特性，并取肿瘤行常规病理切片及CEA、细胞角蛋白、Ki67免疫组化检测，将原代肿瘤和移植瘤进行EGFR 18-21外显子和K-Ras 12，59外显子基因检测，采用流式细胞仪检测移植瘤细胞的细胞周期。

**结果:**

本研究进行了11例肺癌组织的NOD/SCID小鼠和裸鼠建模，建成3例可多次成功传代的腺癌、小细胞肺癌和鳞癌模型。传代移植成功率高。荷瘤小鼠生长情况良好，生存期长。各代移植瘤模型的组织病理学及免疫组化表型、*EGFR*和*K-Ras*基因检测均与来源肺癌组织相一致。移植瘤细胞周期中S期延长，提示瘤细胞有增殖活性。

**结论:**

本研究在国内首次利用新鲜的完整肺癌组织建成了荷肺癌NOD/SCID小鼠及裸鼠模型，并传代移植于裸鼠，建模成功率为27%。移植瘤较好地保留了人原发肺癌的恶性特征及组织病理学、生物学特性，是一种接近人体的肺癌模型，可为肺癌研究提供良好的实验平台。

## Abstract (en)

**Background and objective:**

With the ongoing need to improve therapy for lung cancer, there has been an increasing interest in the development of reliable preclinical models to test novel therapeutics. The aim of this study is to establish a patient-derived lung cancer xenograft model in mice and to observe the biological characteristics of xenografts.

**Methods:**

Surgically resectected tumor specimens from patients with lung cancer were implanted in the subcutaneous layer of the NOD/SCID mice. Cancer specimens of percutaneous lung biopsy by CT fluoroscopy were implanted into the subrenal capsule of nude mouse. The subcutaneous carcinoma was surgically removed when it grew to approximately 1.0 cm in diameter, and then re-transplanted into new nude mice. The growth process of transplanted tumor was observed. Expression of CEA, cytokeratin, and Ki67 were detected by immunohistochemistry. Mutations in the exons 18-21 of EGFR and exons 12, 59 of K-ras of primary and xenograft tumors were examined. The cell cycle of xenograft tumor cells was analyzed by flow cytometry.

**Results:**

Eleven cases were conducted for NOD/SCID mice and nude mice modelling. The patient-derived lung cancer xenografts have been established successfully, and the tumor could be passed to new nude mice, including No 2 model (adenocasinoma), No. 3 model (small cell lung cancer), and No. 5 model (squamous cell cancer). High homogeneity was found between xenograft tumors and human lung cancer in histopathology, immunohistochemical phenotype, and EGFR, *K-ras* mutation status. The S-phase fraction of xenograft cell cycle was prolonged, which indicated that the xenografts remains highly proliferated.

**Conclusion:**

The xenotransplantation models established for patient-derived lung cancer in immune deficient mice. The success rate is 27%. This model system displayed the biological characteristics of human lung cancer, suggesting that it may provide a stable, reliable, and useful animal model in human lung cancer research.

世界卫生组织最新数据表明，到2010年，癌症将成为导致人类死亡的第一病因。在我国第3次居民死亡原因抽样调查中，肺癌更是取代肝癌成为我国首位恶性肿瘤的死亡原因^[1]^。但目前，肺癌的治疗效果仍差强人意。非小细胞肺癌(non-small cell lung cancer, NSCLC)患者的5年生存率仅为10%-15%，小细胞肺癌(small cell lung cancer, SCLC)则仅为5%。为深入了解肺癌的生物学特点及对治疗的应答，需要模拟出与人类肺癌病因、发病机制、发展过程及治疗相似的动物模型。良好的动物模型应能最大限度地模拟人体内环境，而长久以来利用细胞系建立肿瘤模型的弊端明显，研究者已开始认识到组织学完整的瘤组织块与瘤细胞悬液相比是更为理想的移植材料。但目前用新鲜获得的肿瘤组织块作为瘤源建模的研究尚为数不多。2000年Perez-Soler等^[2]^将100例新鲜肺癌组织块植入裸鼠皮下，建模成功率为34%(34/100)，2008年Iduna等^[3]^将102例肺癌标本移植入NOD/SCID小鼠皮下，并传代移植至裸鼠，建模成功率为25%。国内周伟红等^[4]^建立的肺腺癌裸鼠模型，首代的移植成功率是33%，其后传代移植成功率是100%，尚未见可稳定传代的荷肺癌的NOD/SCID小鼠模型的报道。本研究在国内首次利用新鲜的完整肺癌组织建成了荷肺癌NOD/SCID小鼠及裸鼠模型，并传代移植于裸鼠，建模成功率为27%。移植瘤较好地保留了人原发肺癌的恶性特征及组织病理学、生物学特性，是一种接近人体的肺癌模型，可为肺癌研究提供良好的实验平台。

## 1 材料和方法

### 1.1 材料

#### 1.1.1 肿瘤标本来源

本研究的肿瘤标本来源于广东省人民医院手术患者，均为初诊，均有明确病理诊断。原代移植肿瘤组织(F0)直接来源于新鲜标本。1例小细胞肺癌组织标本取自CT引导下经皮肺穿刺活检术。其余10例标本均来自外科切除的原发性肺癌组织，于术中无菌即时获取。

#### 1.1.2 实验动物

NOD/SCID小鼠购自中山大学实验动物中心。雌雄兼用，周龄6周-8周，体重20 g -27 g。BALB/C裸鼠购自北京维通利华实验动物技术有限公司。雌雄兼用，周龄6周-8周，体重18 g-21 g。小鼠均由中山大学实验动物中心在无特定病原体(specified pathogen-free, SPF)条件下饲养。

### 1.2 方法

#### 1.2.1 人肺癌原代皮下移植NOD/SCID小鼠模型的建立

每例肿瘤标本剪成若干个3 mm×5 mm×5 mm的小块备用。NOD/SCID小鼠常规消毒，将肿瘤组织块植入右侧前肢肩背部皮下。每例接种3-5只小鼠，须在肿瘤标本离开人体后1 h内完成接种。此为第1代荷肺癌小鼠(下称F1)。

#### 1.2.2 肺癌皮下移植裸鼠模型的建立

当NOD/SCID小鼠皮下肿瘤生长至大于1cm^3^后，剪开皮肤，剥离皮下肿瘤，部分冻存和10%福尔马林固定备用，部分瘤块作为传代移植的组织来源。取4-5只裸小鼠，按2.1所述方法，建立第2代荷肺癌小鼠(下称F2)。待裸鼠皮下肿瘤长大至直径为1 cm-1.5 cm时取出瘤组织，重复上述手术，进行肿瘤传代，建立第3、4、5代荷肺癌小鼠(下称F3、F4、F5)。

#### 1.2.3 人肺癌原代肾包膜下移植NOD/SCID小鼠模型的建立

肾包膜穿刺接种：将肿瘤穿刺标本剪成约20 mm^3^的碎组织，填入套管针尖端内；裸鼠予0.01 mL/g 4.3%水合氯醛溶液腹腔注射麻醉；摆左侧卧位，固定，消毒；取右侧腹纵切口入腹；托出右侧肾脏于切口外暴露；穿刺部位为肾盂相应的肾包膜；进针后推入针芯，将组织送入肾包膜内；将右肾放回腹腔恢复原位；缝合腹壁，结束手术。术后继续饲养，自由进食。

#### 1.2.4 体内成瘤生长曲线的测定

每日观察荷瘤小鼠全身状况、活动情况，扪查移植瘤生长情况，自接种后4天起每隔3天-4天称量体重并用游标卡尺测量移植瘤体的长径(L)、短径(W)1次，取每例各小鼠肿瘤长短径平均值，按公式V=1/2(L×W^2^)计算移植瘤平均体积。并绘制肿瘤生长变化曲线。

#### 1.2.5 移植瘤的病理学检查

处死小鼠后肉眼观察移植瘤形态、质地、活动度、播散范围及肺、肝、脾、肾等脏器以及淋巴结转移情况。移植瘤及小鼠主要脏器经10%福尔马林固定，石蜡切片(4 μm)，HE染色作常规病理检查。

#### 1.2.6 免疫组织化学检测

进行移植瘤组织Ki67、CEA、细胞角蛋白免疫组织化学的检测：将石蜡包埋的瘤块进行4 μm厚连续切片；60 ℃烘片4 h；切片置于二甲苯40 min以脱蜡，脱蜡后的切片依次置于浓度为100%、95%、80%、75%的梯度酒精中各10 min逐级水化，最后置于蒸馏水；高温高压修复；3%H_2_O_2_室温浸泡10 min；PBS漂洗3次，每次5 min；每张切片各滴加封闭用10%正常山羊血清，室温下孵育30 min，甩去血清；每张切片滴加一抗(鼠抗人Ki67、CEA、细胞角蛋白等，3号病例标本予细胞角蛋白、Syn、NSE、CgA、CD56、TTF1)，平放于湿盒内，4 ℃孵育过夜；室温下复温30min，PBS漂洗三次，每次5 min；滴加二抗，37 ℃孵育30 min；PBS洗涤，5 min×3次；0.05%DAB显色5 min-10 min，在镜下观察显色情况，显色充分后终止着色；苏木素复染；水洗返蓝；梯度酒精至无水酒精脱水，二甲苯透明；中性树胶封片。显微镜下观察并摄像。结果判定：阳性染色为棕黄色颗粒。Ki67阳性表达定位于细胞核，CK7、CK(Pan)阳性表达定位于细胞浆，CEA阳性表达定位于细胞膜和细胞浆。以TBS代替一抗作为阴性对照，阳性对照为已知阳性的标本。

#### 1.2.7 流式细胞仪检测肿瘤细胞细胞周期

将新鲜肿瘤组织剪碎成直径 < 0.5 mm的小块，筛网过滤后离心制成单细胞悬液。按常规方法用流式细胞仪检测细胞周期。以S期细胞百分比(S-phase fraction, SPF)表示细胞的增殖活性。SPF指G_0_/G_1_峰与G_2_/M峰之间的曲线下的面积占整个直方图面积的百分比。SPF=S/(G_0_/G_1_+S+G_2_/M)×100%。

#### 1.2.8 DNA测序-突变分析

从移植瘤组织提取基因组DNA，行EGFR exon18、19、20、21及*K-ras*基因的检测。

### 1.3 统计学处理

采用SPSS 13.0软件进行统计分析，数据以均数数据以Mean±SD或百分率表示，以*P* < 0.05为有统计学差异。

## 2 结果

### 2.1 小鼠皮下及肾包膜下移植、传代情况

小细胞肺癌组织标本取自CT引导下经皮肺穿刺活检术，其余10例组织标本均来自外科切除的原发性肺癌组织。共11例中，男9例、女2例；腺癌6例、鳞癌3例、小细胞癌1例、大细胞癌1例。病例年龄平均61岁。

10例外科切除标本每例皮下移植NOD/SCID小鼠3-5只，共41只。其中，第2号病例建成为第2号(腺癌)模型，第5号病例建成为第5号(鳞癌)模型。见图 1。

**1:**
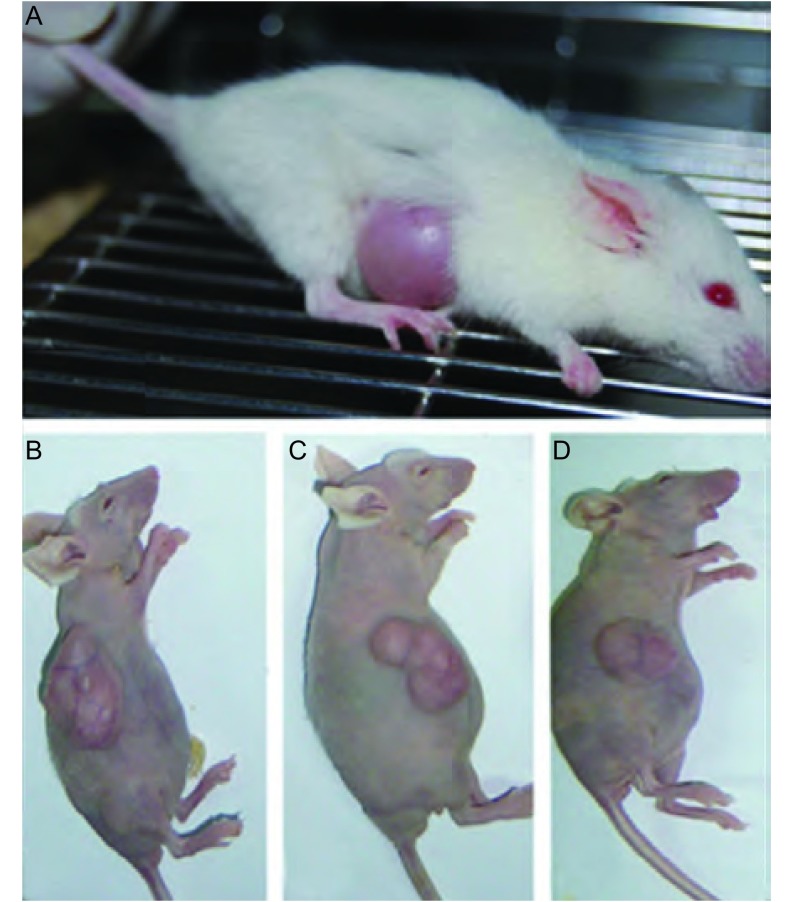
各代荷肺癌小鼠。A：F1荷肺癌NOD/SCID小鼠；B、C、D：F2、F3、F4荷肺癌裸小鼠。 Immunnodeficient mice bearing tumors. A: The first generation transplanted tumor model; B: The second generation transplanted tumor model; C: The third generation transplanted tumor model; D: The fourth generation transplanted tumor model.

**2:**
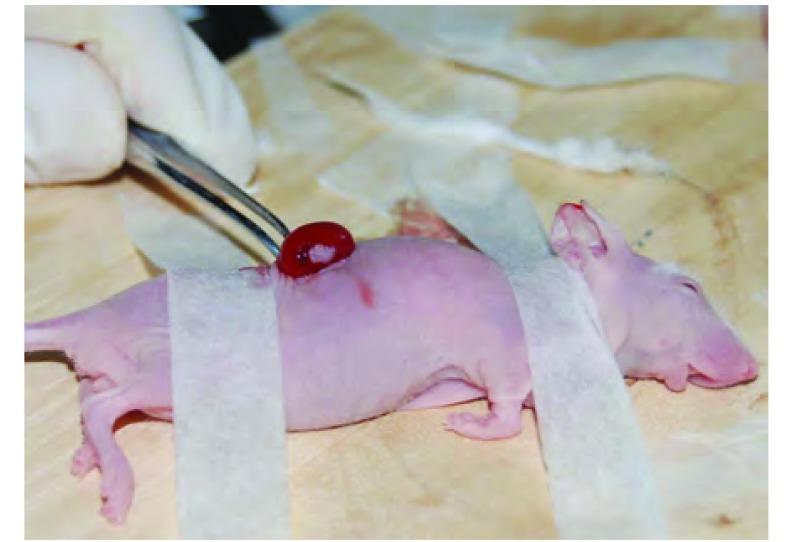
取出荷瘤肾 Removing kidney with tumor

1例(第3号病例)经皮肺穿刺活检术标本因标本量极少，故进行裸小鼠右肾包膜下移植。裸小鼠在移植后第45天取出右肾，经解剖学和组织学证实有肿瘤移植生长。其后在裸小鼠间皮下传代移植。建成为第3号(小细胞肺癌)模型。

### 2.2 小鼠各脏器转移情况NOD/SCID小鼠和裸小鼠解剖后未发现主要脏器有转移情况。

### 2.3 人肺癌模型生长特性

11例肺癌标本，建模成功率为27%(3/11)。F1移植瘤总体成功率69%(29/42)，F2总体成功率59%(16/27)，F3成功率73%(19/26)，F4成功率100%(13/13)，F5成功率97%(32/33)，F6成功率100%(4/4)。

第2号(腺癌)模型F1共4只NOD/SCID小鼠，4个移植瘤均生长良好，F1移植成活率为100%(4/4)。自F2起，皮下移植瘤接种于裸小鼠皮下，目前已传至F6。F2—F6共42只裸小鼠，其中37个移植瘤生长良好，传代移植成活率88.1%。生长曲线见图 3。

**3:**
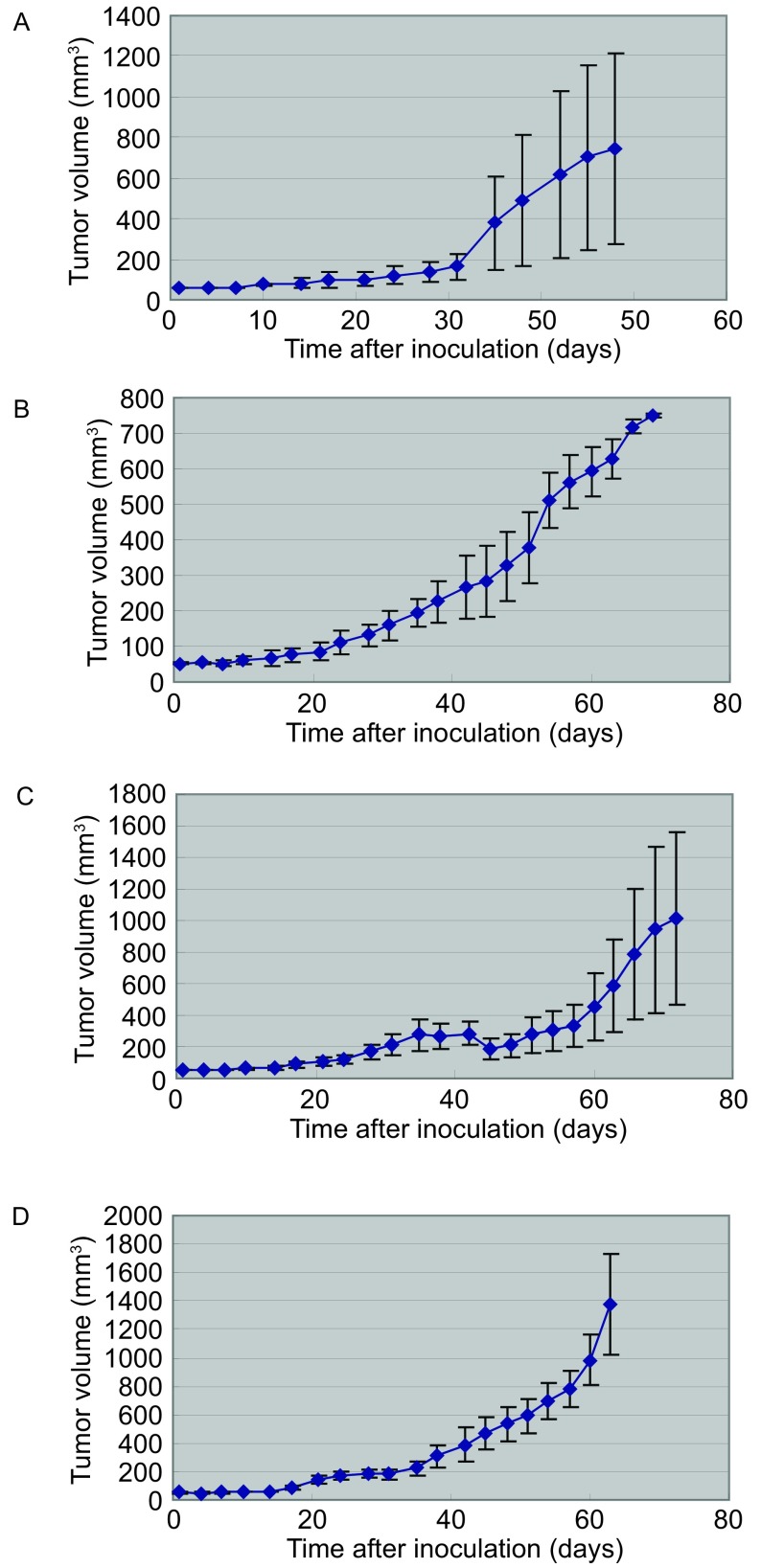
第2号模型（腺癌）小鼠移植瘤生长曲线。A：F1肿瘤生长曲线；B：F2肿瘤生长曲线；C：F3肿瘤生长曲线；D：F4肿瘤生长曲线。 The growth curves of the transplanted tumors after inoculation of model 2. A: The first generation transplanted tumor model; B: The second generation transplanted tumor model; C: The third generation transplanted tumor model; D: The fourth generation transplanted tumor model.

第5号(鳞癌)模型F1共5只NOD/SCID小鼠，5个移植瘤均生长良好，F1移植成活率为100%(5/5)。自F2起，肿瘤接种于裸小鼠皮下，目前已传至F5。F2-F5共24只裸小鼠，其中22个移植瘤生长良好，传代移植成活率91.7%。

### 2.4 移植瘤大体形态

皮下移植瘤外观呈球形或结节状，于表面可见血管纹理。解剖后见瘤体呈乳白色，瘤体被覆不完整结缔组织包膜，毛细血管丰富。

### 2.5 瘤源和移植瘤光镜检查

光镜下可见移植瘤细胞弥漫分布，核大深染，核圆形、卵圆形或不规则形，核染色质不均匀，胞浆少，偶见核仁，可见病理核分裂。与来源肺癌组织形态学特征一致(图 4)。

**4:**
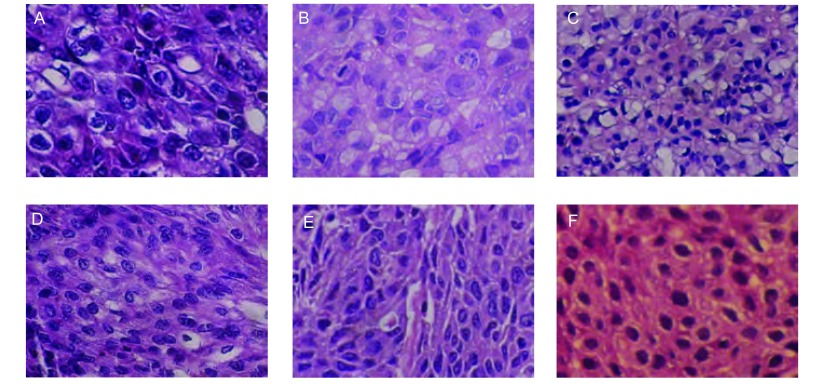
瘤源和移植瘤HE染色图。A：第2号模型F0肿瘤（×200）；B：第2号模型F1肿瘤（×200）；C：第2号模型F4肿瘤（×100）；D：第5号模型F0肿瘤（×200）；E：第5号模型F1肿瘤（×200）；F：第5号模型F4肿瘤（×200）。 Haematoxylin Eosin staining shows histopathologic similarity between the original human tumors (A and D) and their corresponding xenografts in model 2 (B and C) and model 5 (E and F) (×200). A: original tumor of model 2; B: F1 xenograft developed in NOD/SCID mouse of model 2; C: F4 xenograft developed in nude mouse of model 2; D: original tumor of model 5; E: F1 xenograft developed in NOD/SCID mouse of model 5; F: F4 xenograft developed in nude mouse of model 5.

### 2.6 移植瘤流式细胞仪分析

第2号模型(腺癌)移植瘤细胞流式检测：G_1_%=82.6%，G_2_%=2.7%，S%=14.7%(图 5A)。

**5:**
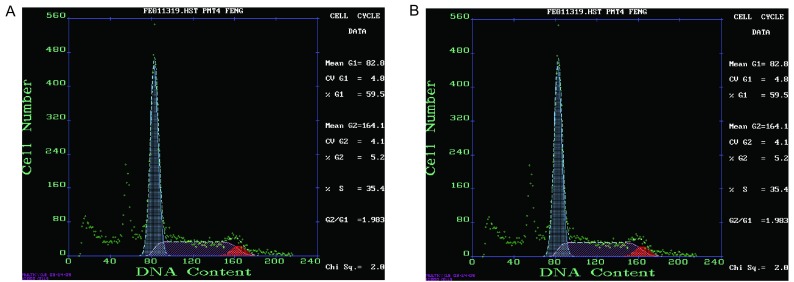
第2号（A）（腺癌）和第5号（B）模型（鳞癌）移植瘤细胞周期图 Cell cycle of No. 2 (A) and No. 5 (B) model xenograft tumor cells

第5号模型(鳞癌)移植瘤细胞流式检测：G_1_%=59.5%，G_2_%=5.2%，S%=35.4%(图 5B)。

通过流式细胞仪检测细胞，提示移植瘤细胞的S期延长，表明移植瘤细胞增殖活性高、分裂期的细胞数目多、生长快。

### 2.7 瘤源和移植瘤肿瘤标志物表达

各模型瘤源和移植瘤大多数标本的CEA、CK 7、CK AE1/AE3均阳性表达(图 6)。

**6:**
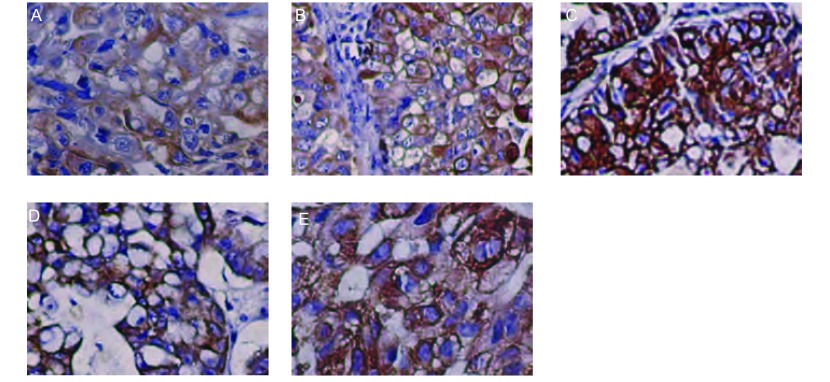
第2号模型瘤源及各代移植瘤CK AE1/ AE3染色图片（×200）。A：F0瘤源；B：F1移植瘤；C：F2移植瘤；D：F3移植瘤；E：F4移植瘤。瘤源和移植瘤CK AE1/AE3均为阳性表达。 Expression of cytokeratin in original human tumor and their corresponding xenogratfs (IHC×200). The cytokeratin staining was positive in both the original tumor (F0, A) and xenografts F1, B; F2, C; F3, D; F4, E) in model 2.

### 2.8 瘤源和移植瘤EGFR18、19、20、21外显子和*K-Ras*12、59外显子基因的检测

对第1-11号瘤源和移植瘤进行EGFR18、19、20、21外显子和*K-Ras*12、59外显子基因的检测：结果提示第2号(腺癌)瘤源和移植均为EGFR野生型，K-Ras G12V突变型；第4号(腺癌)瘤源和移植瘤均为EGFR 21外显子L858R突变，K-Ras野生型；其他各瘤源和移植瘤均为EGFR、K-Ras野生型。提示移植瘤的DNA序列和瘤源保持一致(图 7)。

**7:**
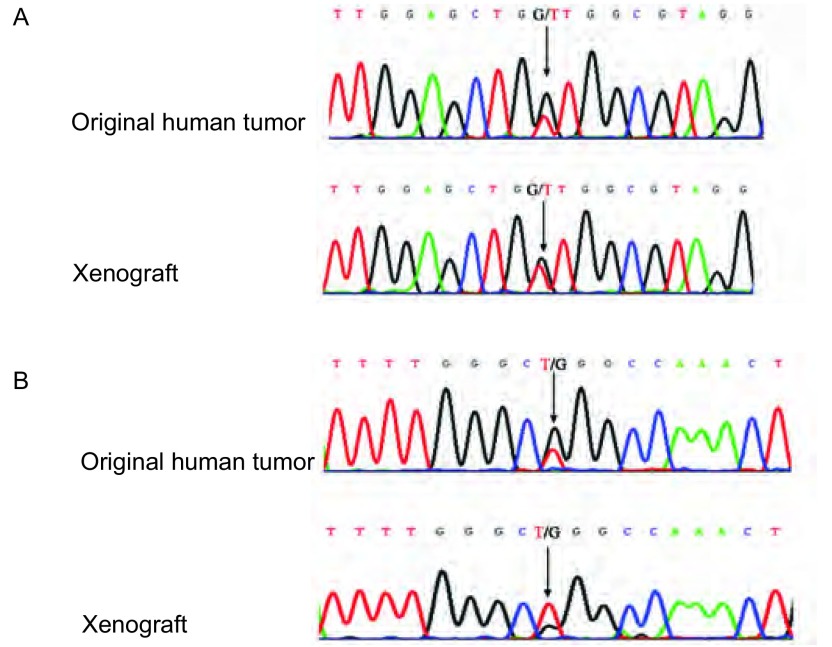
第2号和第4号模型*EGFR*和*K-RAS*突变测序图。A：第2号模型原代肿瘤和移植瘤均为K-ras G12V突变(G→T)；B：第4号模型原代肿瘤和移植瘤均为EGFR 21exon L858R突变(T→G)。 DNA analysis for *EGFR* and *K-RAS* mutations in model 2 and model 4. Mutations in K-ras and EGFR were detected in model 2 (G12V) and model 4 (21exon L858R), including original tumors and xenografts.

## 3 讨论

### 3.1 完整肿瘤组织块移植模型的优点

将肺癌患者的新鲜癌组织接种动物建立的原代动物模型是重要的肺癌研究工具，除了可以作为转化性和基础研究的模型，还可以用于评价药物的疗效，从而为肺癌的个体化用药带来新的评价工具^[5]^。这种移植方式最大程度地保存了肿瘤的微环境，包括肿瘤细胞、浸润的淋巴细胞、成纤维细胞、细胞外间质和脉管系统^[6, 7]^。较之前用肿瘤细胞系悬液建模的方法，可以更深入研究肿瘤—间质之间的复杂关系和相互作用，比如肿瘤细胞和肿瘤相关细胞是如何互相激活和促进侵袭的^[6-11]^，而且只有轻微的移植物抗宿主反应。移植瘤还能保留人体原代肿瘤的病理形态学、细胞生物学机能、染色体特征、肿瘤标志物等特点, 成瘤率和转移率较细胞系模型均明显提高，能更准确地反映肿瘤的特点和干预措施的影响，因而是目前最为理想的肿瘤模型。造成这种差异的主要原因是：移植用的瘤细胞悬液经过消化酶处理，肿瘤的原有结构被破坏，细胞表面结构也遭到破坏而发生变化，导致肿瘤生物学行为和自然属性改变，使肿瘤细胞之间的协同性降低，影响了其恶性程度的表达^[12]^，进而影响了肿瘤细胞的生长与转移。

这种模型还有一个明显的优势，就是有利于个体化抗癌方案的研究。我们建立的这种人-鼠模型，就是很好的“一对一”的个体化模型，即在小鼠体内最大程度地“复制”标本来源的患者的荷瘤环境，以供研究针对此患者的个体化治疗方案，较之利用在体外反复传代培养的细胞系建立的“广谱”模型，针对性大大加强。还可以在传代的小鼠体内取得源源不断的新鲜肿瘤标本，用来建立肿瘤细胞系，进行多种抗瘤的体外实验及其他各项检测。

以后，我们可以考虑这样一种建模方法：早、中期的适于外科手术切除的患者，可以在术中获取足量的移植瘤瘤源接种于小鼠皮下，而晚期患者失去手术机会或如本实验中的小细胞肺癌患者，则可以从原发或转移灶的穿刺活检术中获取少量瘤源接种于小鼠肾包膜下，这样就解决了部分患者难以取得足够的建模标本量的问题。

但这种模型建模时间长，对动物外科学的要求较高，原代移植成功率较低，往往要重复多次，或移植数例方可成功。我们的建模成功率是27%。以后还要继续改进方法，以提高临床实用性。

### 3.2 实验动物的选择

建立肿瘤模型首先必须解决宿主动物的免疫排斥反应。因此免疫缺陷动物是异种移植的最佳受体。NOD/SCID小鼠是T、B和NK三种细胞功能缺陷的动物，它是由SCID小鼠与具有NK细胞功能缺陷的、循环补体缺乏、抗原递呈细胞分化及功能不良特点的NOD/Lt品系回交而得到的免疫缺陷动物。这种三联免疫缺陷动物和两种细胞(如T、B细胞功能缺陷的SCID小鼠)或一种细胞(如T细胞功能缺陷的裸鼠)功能缺陷的免疫缺陷动物比较，其免疫力更为低下，因而也更容易接受异种移植^[13]^，目前被认为是最理想的移植瘤受体动物^[14]^。在我们的研究中，外科切除术中获得的新鲜肺癌标本，首先接种于NOD/SCID鼠皮下，再传代于裸鼠。这是因为NOD/SCID鼠的深度免疫缺陷使其更易于接受异体移植物，更利于原代肿瘤的生长，而F1肿瘤在NOD/SCID鼠体内的生长过程中要经受多种组织内环境的“压力”，这种体内的压力选择，使肿瘤细胞群体中的高侵袭克隆得以优势增长，F2以后各代肿瘤恶性程度相对更高，肿瘤生长趋向稳定，故易于在仅T淋巴细胞成熟缺陷的裸鼠体内生长，我们的研究结果也证明传代后在裸鼠体内成瘤率很高。这样，不仅节约了实验成本(NOD/SCID鼠较裸鼠昂贵)，而且因为裸鼠生存期较NOD/SCID鼠长，可以用来进行药敏试验等观察期较长的实验。

### 3.3 移植瘤模型的病理学特点、生物学特性

移植瘤在小鼠皮下连续传代，却保持原发肿瘤的组织学特点，这是人肿瘤小鼠移植模型能反映人类肿瘤生物学特点的基本条件^[15]^。在我们所建的模型中，小鼠皮下移植瘤模型的组织病理学、免疫组化表型等均与人体来源的肿瘤细胞相一致，*EGFR*和*K-Ras*外显子基因的检测亦提示移植瘤的DNA序列和瘤源保持一致，表明移植瘤保持了原代肿瘤的恶性表型和生物学特性，并在传代中稳定表达。大多数模型移植瘤都表达Ki67，可见移植瘤增殖活跃，符合肿瘤的生物学特性。细胞周期的结果表明移植瘤细胞增殖活性高、分裂期的细胞数目多、生长快。

大多数研究认为，移植瘤的生长速度大约在F3--F4代开始稳定，在本实验中建成的第2、3、5号模型中，F4移植瘤大约在传代30天后生长略有加速(图 4)。但生长加速的趋势并不十分明显，分析原因可能有以下几点：①由于小鼠的淋巴系统免疫缺陷，抵抗力较差，长时间会增加感染和其它疾病的风险；②受空间限制的影响，小鼠不能为移植瘤体的生长提供足够的空间，也不能为移植瘤的增大提供足够的营养；③少部分小鼠可能发生渗漏现象，即随着鼠龄增长，小鼠T、B淋巴细胞功能部分恢复。这些原因都可能影响实验结果。但本研究中建成的模型例数还太少，不足以充分观察移植瘤的生长情况，还需进一步深入研究。
